# Effect of Early Administration of Clarithromycin or Azithromycin on Symptoms of Pertussis in Infants

**DOI:** 10.3390/antibiotics14030279

**Published:** 2025-03-08

**Authors:** Alberto Eugenio Tozzi, Ileana Croci, Francesco Gesualdo, Carlo Federico Perno, Giulia Linardos, Alberto Villani, Luisa Russo, Ilaria Campagna, Diana Ferro, Elisabetta Pandolfi

**Affiliations:** 1 Predictive and Preventive Medicine Research Unit, Bambino Gesù Children’s Hospital, IRCSS, 00165 Rome, Italy; albertoeugenio.tozzi@opbg.net (A.E.T.); f.gesualdo@gmail.com (F.G.); pandolfi.elisabetta@gmail.com (E.P.); 2 Unit of Microbiology and Diagnostic Immunology, Bambino Gesù Children’s Hospital, IRCSS, 00165 Rome, Italy; carlofederico.perno@opbg.net (C.F.P.); giulia.linardos@opbg.net (G.L.); 3 General Pediatric and Infectious Disease Unit, Bambino Gesù Children’s Hospital, IRCSS, 00165 Rome, Italy; alberto.villani@opbg.net; 4 Health Directorate, Bambino Gesù Children’s Hospital, IRCSS, 00165 Rome, Italy; luisa.russo@opbg.net; 5 Pediatric Semi-Intensive Care Unit, Bambino Gesù Children’s Hospital, IRCSS, 00165 Rome, Italy; ilaria.campagna@opbg.net

**Keywords:** pertussis, clarithromycin, azithromycin, cough, infants

## Abstract

**Background:** A resurgence of pertussis has been observed in several geographic areas in the post-COVID-19 era. Macrolides are the first-choice antibiotics for the treatment of pertussis. Limited data exist on the impact of the early administration of clarithromycin or azithromycin on infants’ pertussis symptoms. **Methods:** This retrospective cohort study analyzed infants enrolled in an enhanced surveillance program for pertussis at a single Italian clinical reference center between 2015 and 2020. All cases were laboratory-confirmed. This study compared outcomes based on the timing of macrolide antibiotic treatment: early administration (within 7 days of cough onset) versus late administration (8 days or later). Key outcomes included cough duration, symptom frequency, and complication rates. **Results**: We studied 148 infants with confirmed pertussis. The median duration of coughing was 14 days in infants with early administration and 24 days in those with late administration. The occurrence of symptoms differed for apnea (62.6% for early administration; 84.6% for late administration). In a multivariable Cox model, the duration of the cough was lower in infants receiving antibiotics within 7 days from the beginning of the cough compared with those starting later (HR = 0.36, 95% CI: 0.25–0.53, *p* < 0.001). Clarithromycin was associated with a shorter duration of coughing (HR = 0.42, 95% CI: 0.19–0.92, *p* = 0.030) independently from other factors. Regarding the occurrence of symptoms, children receiving antibiotics later were three times more likely to experience apnea compared to those treated early (*p* = 0.008). **Conclusions:** Early treatment with clarithromycin or azithromycin for infants with pertussis improves clinical symptoms. Clarithromycin may be more effective than azithromycin in shortening coughing. The early administration of antibiotics may also help prevent the spread of disease during the resurgence of pertussis and should be considered regardless of the laboratory confirmation, while taking into account the potential side effects of an unnecessary therapy.

## 1. Introduction

The post-COVID-19 era is characterized by a disruption in the usual epidemiology of infectious diseases and the reemergence of several vaccine-preventable diseases [1,2]. This phenomenon is partly due to a decreased population immunity to other pathogens—often referred to as immunity debt [3]—associated with non-pharmaceutical interventions, and a decline in routine immunization coverage [4,5].

As with other transmissible diseases, a resurgence of pertussis has been observed in several countries since 2022, following a period of low circulation [6,7]. In the United States, pertussis cases in 2024 rose to six times the number recorded in 2023 [8]. In the EU/EEA, over 25,000 cases were reported in 2023, with more than 32,000 cases recorded between January and March 2024, the highest incidence occurring in infants below one year of age [9], particularly in Spain [10], Finland [11], Italy [12], Denmark [13], France [14], and the UK [9]. A resurgence of pertussis has also been observed in Israel [15], Australia [16], and China [7].

Macrolides, which inhibit the protein synthesis of bacteria binding to the 50S subunit of the bacterial ribosome, are the first-choice antibiotics for pertussis treatment [17]. These antibiotics remain effective in the EU and the USA, although macrolide-resistant strains of *B. pertussis* have been reported in other regions [18], including Europe [11,14]. The appropriateness of antibiotic treatment has potential implications in the emergence of antibiotic resistant strains of *B. pertussis*. Historically, erythromycin was the most-used macrolide for pertussis treatment, but azithromycin and clarithromycin have been recommended due to their improved absorption, comparable efficacy, and better tolerability compared to erythromycin [19,20]. A systematic review concluded that there is no evidence that antibiotic treatment of pertussis affects the clinical course of the disease [21]. Some studies suggested that early administration of antibiotics may ameliorate the clinical picture; however, these studies did not specify the type of antibiotic included in the analysis and did not focus solely on infants [22,23]. Despite the lack of definitive evidence, early antimicrobial therapy is recommended if the clinical history strongly suggests pertussis or if the patient is at high risk of severe or complicated disease (e.g., an infant) [17]. To our knowledge, no study has yet analyzed the effect of the timing of initiation of treatment with azithromycin or clarithromycin on pertussis symptoms in infants. Therefore, we investigated whether early treatment with azithromycin or clarithromycin can reduce the cough duration in patients with pertussis below one year of age compared with late treatment.

## 2. Results

A total of 165 out of 599 infants with respiratory symptoms were confirmed to be infected by *B. pertussis*. Among these confirmed cases, 160 children (96.97% of positive cases) received an antibiotic treatment. Of those, 10 infants were treated with beta-lactam antibiotics (amoxicillin/cephalosporin). For two infants, the antibiotic was unknown.

The remaining 148 infants received clarithromycin (91.9%) 15 mg/kg/day or azithromycin (8.1%) 10 mg/kg/day and were included in the analysis.

The study population is described in Table 1 by the timing of treatment administration. Among participants, 40% were too young to be vaccinated. Of the eligible infants, 28.7% had not received any vaccine doses. Additionally, 24.7% had received one dose, and 6.7% had received two doses. None of the mothers of included patients had been immunized against pertussis during pregnancy. A significant difference between the two groups was observed only in cough duration, with the early administration group experiencing a shorter median cough duration of 14 days (IQR: 8–22) compared to 24 days (IQR: 19–36) in the late administration group (*p* < 0.001).

The frequency of symptoms by timing of treatment initiation is presented in Table 2.

The effect of the timing of treatment initiation on symptom occurrence based on the results of logistic regression analyses is reported in Table 3.

Children who started antibiotic therapy > 7 days after the onset of coughing were three times more likely to experience apnea compared to those who received early treatment (*p* = 0.008). Although other symptoms were more frequent in the late initiation group, these differences were not statistically significant.

**Figure 1:**
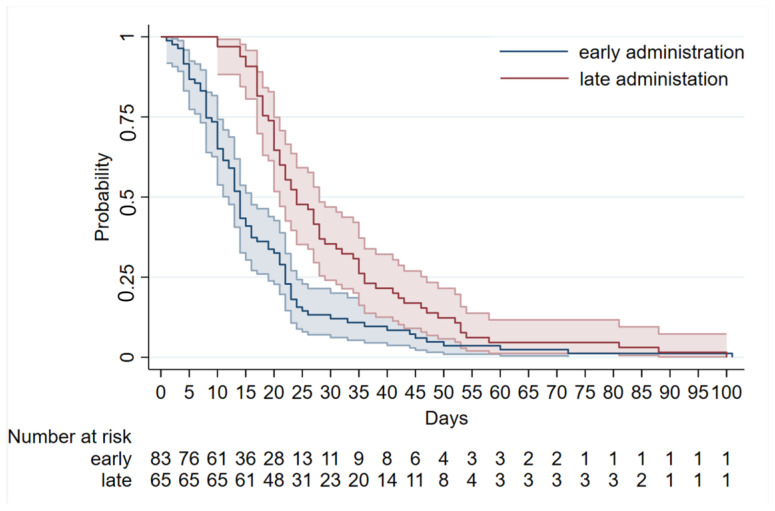
Duration of cough by macrolide timing through Kaplan-Maier curves.

Figure 1 shows the duration of coughing by the time of macrolide initiation using Kaplan–Maier curves. 

Infants who received early treatment had a shorter and more rapidly decreasing duration of coughing compared to those treated later. When the effect of macrolide timing on cough duration was analyzed with the Cox proportional hazards model (Table 4), the hazard ratio for late administration was 0.36 (95% CI: 025–0.53, *p* < 0.001) indicating a significantly longer cough duration compared to early administration. In addition, infants receiving azithromycin had a longer cough duration compared with those receiving clarithromycin. Finally, the duration of antibiotic treatment increased with the duration of coughing. Other covariates did not exhibit statistically significant effects on cough duration, as shown in Table 4.

## 3. Discussion

Our study suggests that early treatment with clarithromycin or azithromycin reduces both the duration of coughing and the occurrence of apnea in infants under one year of age with pertussis. These findings align with results from other studies that examined the effects of various macrolides across different age groups [22,23,24]. Moreover, studies conducted on infants also suggest that early macrolide treatment is associated with a higher survival rate [24,25]. However, the available evidence for infants pertains to erythromycin or other unspecified antibiotics.

Our findings confirm and support the current recommendation of an early initiation of clarithromycin or azithromycin in infants when a diagnosis of pertussis is suspected [17]. Implementing this approach has practical implications.

First, to facilitate early treatment, timely diagnosis—either through laboratory confirmation [26] or based on clinical symptoms [27]—is essential. Second, in high-risk groups such as infants, antibiotic therapy should be considered based on clinical symptoms alone, even if laboratory confirmation is unavailable. Third, the decision to prescribe antibiotics without laboratory confirmation should be considered against the potential impact of unnecessary treatment on antibiotic resistance [28].

In the current context, where pertussis has reemerged, having laboratory confirmation readily available for a rapid response is crucial for proper disease management. However, in many settings this may not be feasible due to the limited availability of reference laboratories [29]. As a result, infants with a cough and other suggestive symptoms of pertussis are often started on antibiotic treatment only after laboratory confirmation [30]. Notably, among the children in our study, 65% had been previously seen by a family pediatrician. Of these, none had received laboratory confirmation of pertussis, and only 54% had been administered antibiotics before accessing the ED.

We also observed that clarithromycin treatment was associated with a shorter duration of coughing compared with azithromycin. We did not identify any intervention study comparing the clinical efficacy of these two antibiotics in the available literature. As our findings may inform clinical recommendations, a randomized controlled clinical trial is needed to confirm this result.

We included antibiotic treatment duration in the multivariable model to account for differing indications on duration of treatment for clarithromycin and azithromycin [17]. Our results indicated that an extended duration of antibiotic treatment was associated with a longer cough duration. This result likely reflects a choice by the physician to continue the treatment due to persistent symptoms, which is a potential outcome in children with pertussis despite microbiological eradication.

Current evidence does not conclusively determine whether liberal use of macrolides is linked to antibiotic resistance in *B. pertussis* [31]. However, while in some countries *B. pertussis* strains resistant to macrolides are commonly reported [32], such resistance started to appear in the EU only recently. Moreover, although azithromycin and clarithromycin are well tolerated, they are not devoid of side effects [17]. While individuals at risk should receive antibiotic treatment early independently from laboratory confirmation, improving the accuracy of clinical diagnosis is advisable.

We have previously shown that a decision tree tool based solely on clinical symptoms can exclude pertussis with a 94% probability [33]. Clinical decision support systems based on large databases of clinical patterns may facilitate an early and accurate clinical diagnosis of pertussis on a large scale, and can support informed decisions regarding antibiotic therapy before laboratory confirmation is obtained.

Immunization during pregnancy is prioritized to prevent pertussis in the most vulnerable group—infants too young to be vaccinated [34]. However, immunization rates among this population remain suboptimal, with significant variations by country [9]. In our study, none of the mothers of the enrolled children had been immunized against pertussis during pregnancy. In regions where immunization rates in pregnancy are low, it is crucial not only to strengthen immunization programs but also to establish appropriate antibiotic treatment strategies for treating infants with suspected pertussis.

Shortening the duration of coughing and preventing apnea could lead to reduced healthcare costs by minimizing the need for extended medical care and hospitalizations [35]. Although early antibiotic treatment is likely to prevent complications of the disease, our sample size was insufficient to conclusively demonstrate this effect. Additionally, early administration of macrolides may help decrease the circulation of the infection, thereby preventing new cases [36].

Our study had strengths. We examined infants treated exclusively with clarithromycin or azithromycin, with a pertussis diagnosis confirmed by laboratory tests at a single reference center. Furthermore, these patients underwent standard screening for clinical symptoms as part of their enrollment in an enhanced surveillance program, which followed a standardized protocol.

A potential bias in assessing the impact of early antibiotic treatment on symptom duration in observational studies could stem from the pattern of care-seeking behavior, which has been linked to various confounding variables [37]. This bias may be prominent in studies based on public health surveillance in which factors influencing the likelihood of accessing medical care have not been accounted for. In our study, we collected the onset date of symptoms for all cases in our cohort through structured interviews with mothers, enabling precise calculation of cough duration. Additionally, we gathered information on potential confounders affecting access to healthcare resources, such as the season, the family’s social status, and distance from the hospital, and corrected for them in multivariate models.

On the other hand, the limited number of observations in this study may have restricted our ability to accurately assess the effects of early antibiotic administration on relatively rare complications. Additionally, the observational design of this study exposed it to recall bias when collecting information through interviews. While it would be ethically questionable to conduct a randomized clinical trial on the timing of antibiotic administration, we implemented strict timing protocols for the interviews to minimize recall bias. Furthermore, this study only included infants who accessed the ED at a hospital facility. Consequently, we cannot automatically generalize these results to patients with milder symptoms who are managed within a primary care setting. Finally, our study did not include pertussis cases from the broader community.

In conclusion, early administration of clarithromycin or azithromycin in infants with pertussis may help shorten the duration of coughing and decrease the occurrence of apnea. Moreover, clarithromycin may be more effective in shortening cough duration compared with azithromycin. Although we could not measure the impact of treatment timing on complications or access to hospital care, an early initiation of treatment potentially has a significant impact on the quality of life of affected families, especially during the re-emergence of the disease. Given the high transmissibility of pertussis, early treatment can also help limit its spread. Performing a laboratory confirmation test early in the disease course is advisable, although relying on clinical symptoms alone for antibiotic prescription should be considered, especially in infants. These results may inform clinical practice and help support clinical decisions on antibiotic treatment in infants with symptoms of pertussis. Further studies should confirm differences in the clinical efficacy of clarithromycin vs. azithromycin, monitor the potential emergence of macrolide-resistant strains of *B. pertussis* in specific geographic areas, and adapt local antibiotic strategies accordingly.

## 4. Materials and Methods

### 4.1. Study Population

This was a retrospective cohort study of patients enrolled in an enhanced hospital surveillance program for pertussis at the Bambino Gesù Children’s Hospital in Rome, Italy, between August 2015 and June 2020 [38].

In the original study, the study population consisted of infants under one year of age hospitalized with respiratory symptoms. Upon hospitalization, and after obtaining informed consent, we collected demographic information, signs, symptoms, and treatment information through a standardized questionnaire. Moreover, an RT-PCR for *B. pertussis* and other respiratory pathogens was performed on nasopharyngeal aspirate. Subsequently, parents of enrolled infants were interviewed with a standardized questionnaire at discharge and on weekly phone calls until the end of the cough. The original surveillance study was approved by the Bambino Gesù Children’s Hospital Ethical Committee (protocol n.1064_OPBG_2016).

In the present study, we included infants enrolled in the context of the original surveillance study with an RT-PCR on the nasopharyngeal aspirate positive for *B. pertussis*.

### 4.2. Outcomes

Cough duration was calculated based on the start and end dates as reported by parents during interviews. Complications during hospitalization were defined as the presence of at least one of the following conditions: hypoxia, cerebral hemorrhage, seizures, and superinfection.

### 4.3. Laboratory Confirmation

Nasopharyngeal aspirates were processed following a standardized protocol. *B. pertussis* was detected using a real-time PCR kit specifically targeting the IS481 gene (Bordetella R-gene assay, Argene, Biomerieux, Marcy L’etoile, France).

### 4.4. Statistical Analysis

Continuous variables were reported as medians and interquartile ranges (IQRs), and categorical variables were tabulated as frequencies and percentages. Continuous and categorical data were compared across groups using Wilcoxon–Mann–Whitney and chi-square tests. We observed that the median time between the onset of coughing and antibiotic start was 7 days. We therefore categorized included children into two groups: those with macrolide administration within 7 days after the onset of coughing (early) and those with macrolide administration > 7 days after the onset of coughing (late).

We used logistic regression analysis to estimate the effect of the timing of initiation of macrolide treatment on the occurrence of the following symptoms: apnea, cyanosis, vomiting, and whooping. Moreover, we used Kaplan–Maier curves and a Cox model [39] to assess the impact of the timing of treatment initiation on the duration of the cough. In the logistic regression model and the Cox model, we considered the following variables as potential confounders: child’s age, sex, coinfections (no/yes), exclusive breastfeeding (no/yes), pertussis vaccination (no, one dose, or two doses), complications during hospitalization, smoking in the family, household member with symptoms, season at onset of symptoms (summer vs. other), type of antibiotic (azithromycin or clarithromycin), duration of treatment, presence of socioeconomic problems (no/yes), and the distance in km between a patient’s home and the ED. Missing data were not imputed. Complete case analyses were performed. All data analyses were performed using STATA 17 (Stata Corp., College Station, TX, USA).

## Figures and Tables

**Table 1:** Characteristics of the study population.

	Total (N = 148)	Early Administration ^a^ (N = 83)	Late Administration ^b^ (N = 65)	*p*
Age in months, median (IQR)	2.6 (1.6–3.8)	2.4 (1.3–4.0)	2.7 (1.8–3.5)	0.490
Males, No. (%)	85 (57.4%)	49 (59.0%)	36 (55.4%)	0.656
Season at the onset of symptoms, No. (%)				
Summer	50 (33.8%)	23 (27.7%)	27 (41.5%)	0.078
Other	98 (66.2%)	60 (72.3%)	38 (58.5%)	
Coinfections, No. (%)	76 (51.4%)	47 (56.6%)	29 (44.6%)	0.204
Exclusive breastfeeding, No. (%)	64 (43.2%)	36 (43.4%)	28 (43.1%)	0.971
Smoking in the family, No. (%)	55 (37.2%)	29 (34.9%)	26 (40.0%)	0.527
Household member with symptoms ^c^, No. (%)	106 (77.4%)	57 (73.1%)	49 (83.1%)	0.167
Vaccine, No. (%)				
No	101 (68.2%)	58 (69.9%)	43 (66.2%)	0.790
One dose	37 (25.0%)	19 (22.9%)	18 (27.7%)	
Two doses	10 (6.8%)	6 (7.2%)	4 (6.1%)	
Type of macrolide ^d^, No. (%)				0.177
Azithromycin	12 (8.2%)	9 (10.8%)	3 (4.7%)	
Clarithromycin	135 (91.8%)	74 (89.2%)	61 (95.3%)	
Duration of treatment, median (IQR)	13 (8–14)	13 (7–15)	13 (8–14)	0.787
Socioeconomic problems ^d^, No. (%)	25 (17.0%)	18 (21.9%)	7 (10.8%)	0.073
Distance in km ^e^, median (IQR)	32.8 (13.6–50.6)	34.5 (18.3–57.1)	28.6 (10.8–45.7)	0.101
Complications during hospitalization ^f^, No. (%)	17 (11.5%)	11 (13.3%)	6 (9.2%)	0.446
Length of stay, median (IQR)	6 (4–10.5)	6 (4–13)	6 (4–9)	0.768
Cough duration, median (IQR)	20 (13–28)	14 (8–22)	24 (19–36)	<0.001

^a^ within 7 days after the onset of coughing; ^b^ >7 days after the onset of coughing; ^c^ 11 missing values; ^d^ 1 missing value; ^e^ distance in km between home and ED; 2 missing values; ^f^ at least one of the following conditions: hypoxia, cerebral hemorrhage, seizures, and superinfection.

**Table 2:** Characteristic symptoms of pertussis infection.

	Total (*n* = 148)	Early Administration ^a^ (*n* = 83)	Late Administration ^b^ (*n* = 65)	*p*
Cyanosis, No. (%)	86 (58.1%)	46 (55.4%)	40 (61.5%)	0.454
Vomiting, No. (%)	60 (40.5%)	31 (37.4%)	29 (44.6%)	0.372
Apnea, No. (%)	107 (72.3%)	52 (62.6%)	55 (84.6%)	0.003
Whoop, No. (%)	77 (52.0%)	39 (47.0%)	38 (58.5%)	0.166

^a^ within 7 days after the onset of coughing; ^b^ >7 days after the onset of coughing.

**Table 3:** Effect of timing of antibiotic administration on occurrence of symptoms.

	Late Administration of Macrolide (Ref: Early Administration)					
	Unadjusted			Adjusted ^a^		
	OR	95% CI	*p*	OR	95% CI	*p*
Cyanosis	1.29	0.66–2.49	0.454	1.26	0.59–2.67	0.555
Vomiting	1.35	0.70–2.62	0.372	1.84	0.83–4.09	0.132
Apnea	3.28	1.46–7.35	0.004	3.02	1.26–7.26	0.013
Whoop	1.59	0.82–3.06	0.167	1.69	0.80–3.57	0.169

^a^ Logistic models adjusted for age in months, sex, feeding, smoking in the family, coinfections, households with symptoms, pertussis vaccination, complications during hospitalization, season at onset of symptoms, socioeconomic problems, type of macrolide, duration of treatment, and distance between patient’s home and ED.

**Table 4:** Effect of timing of antibiotic administration on cough duration.

	Cough Duration		
	HR	95% CI	*p*
Late administration of macrolide	0.36	0.25–0.53	<0.001
Age in months	1.07	0.93–1.24	0.346
Females	0.83	0.58–1.18	0.300
Coinfections	0.86	0.57–1.29	0.466
Exclusive breastfeeding	1.34	0.92–1.96	0.129
Complications during hospitalization	0.64	0.33–1.22	0.175
Vaccine (Ref: No)			
One dose	0.74	0.42–1.27	0.274
Two doses	0.73	0.25–2.16	0.566
Smoking in the family	0.80	0.58–1.18	0.247
Household with symptoms	1.35	0.85–2.15	0.206
Season at the onset of symptoms (Ref: Other)			
Summer	0.75	0.50–1.13	0.173
Type of macrolide (Ref: Clarithromycin)			
Azithromycin	0.42	0.19–0.92	0.030
Duration of treatment	0.93	0.89–0.94	<0.001
Socioeconomic problems	1.34	0.83–2.16	0.227
Distance in km	1.00	0.99–1.00	0.379

## Data Availability

The datasets presented in this article are not readily available because GDPR limitations. Requests to access the datasets should be directed to albertoeugenio.tozzi@opbg.net.

## References

[1] Hamson E., Forbes C., Wittkopf P., Pandey A., Mendes D., Kowalik J., Czudek C., Mugwagwa T. (2023). Impact of pandemics and disruptions to vaccination on infectious diseases epidemiology past and present. Hum. Vaccines Immunother..

[2] Baker R.E., Park S.W., Yang W., Vecchi G.A., Metcalf C.J.E., Grenfell B.T. (2020). The impact of COVID-19 nonpharmaceutical interventions on the future dynamics of endemic infections. Proc. Natl. Acad. Sci. USA.

[3] Cohen R., Ashman M., Taha M.-K., Varon E., Angoulvant F., Levy C., Ryback A., Ouldali N., Guiso N., Grimprel E. (2021). Pediatric Infectious Disease Group (GPIP) position paper on the immune debt of the COVID-19 pandemic in childhood, how can we fill the immunity gap?. Infect. Dis. Now.

[4] Treharne A., Murthy B.P., Zell E.R., Jones-Jack N., Loper O., Bakshi A., Nalla A., Kuramoto S., Cheng I., Dykstra A. (2024). Impact of the COVID-19 pandemic on routine childhood vaccination in 9 U.S. jurisdictions. Vaccine.

[5] Lazarus J.V., White T.M., Wyka K., Ratzan S.C., Rabin K., Larson H.J., Martinon-Torres F., Kuchar E., Karim S.S.A., Giles-Vernick T. (2024). Influence of COVID-19 on trust in routine immunization, health information sources and pandemic preparedness in 23 countries in 2023. Nat. Med..

[6] Khalil A., Samara A., Campbell H., Ladhani S.N., Amirthalingam G. (2024). Recent increase in infant pertussis cases in Europe and the critical importance of antenatal immunizations: We must do better…now. Int. J. Infect. Dis..

[7] Liu Y., Ye Q. (2024). Resurgence and the shift in the age of peak onset of pertussis in southern China. J. Infect..

[8] CDC Pertussis Surveillance and Trends. Whooping Cough (Pertussis). Published 11 July 2024. https://www.cdc.gov/pertussis/php/surveillance/index.html.

[9] European Centre for Disease Prevention and Control (2024). Increase of Pertussis Cases in the EU/EEA: 8 May 2024.

[10] Poltorak V., Cabré-Riera A., Martínez-Botías F., López E.B., Romero L.C., Farré M.R.S., Checa M.J., Working Group for surveillance of pertussis in Vallès (2024). Increase of pertussis cases in the Vallès region, Catalonia, Spain, September 2023 to April 2024. Euro Surveill..

[11] Miettinen M., Barkoff A.-M., Nyqvist A., Savolainen-Kopra C., Antikainen J., Mertsola J., Ivaska L., He Q. (2024). Macrolide-resistant Bordetella pertussis strain identified during an ongoing epidemic, Finland, January to October 2024. Euro Surveill..

[12] Poeta M., Moracas C., Albano C., Petrarca L., Maglione M., Pierri L., Carta M., Montaldo P., Venturini E., De Luca M. (2024). Pertussis outbreak in neonates and young infants across Italy, January to May 2024: Implications for vaccination strategies. Euro Surveill..

[13] Nordholm A.C., Emborg H.-D., Nørgaard S.K., Nygaard U., Ronayne A., Nielsen L.B., Søborg B., Andersen P.H., Dalby T. (2024). Pertussis epidemic in Denmark, August 2023 to February 2024. Euro Surveill..

[14] Rodrigues C., Bouchez V., Soares A., Trombert-Paolantoni S., El Belghiti F.A., Cohen J.F., Armatys N., Landier A., Blanchot T., Hervo M. (2024). Resurgence of Bordetella pertussis, including one macrolide-resistant isolate, France, 2024. Euro Surveill..

[15] Stein-Zamir C., Shoob H., Abramson N., Brown E.H., Zimmermann Y. (2023). Pertussis outbreak mainly in unvaccinated young children in ultra-orthodox Jewish groups, Jerusalem, Israel 2023. Epidemiol. Infect..

[16] (2024). Care AGD of H and A. ATAGI 105th Meeting Bulletin—16 and 17 May 2024. https://www.health.gov.au/resources/publications/atagi-105th-meeting-bulletin-16-and-17-may-2024?language=en.

[17] Red Book: 2024 Report of the Committee on Infectious Diseases, 33rd Edition [Paperback] | shopAAP n.d. https://www.aap.org/Red-Book-2024-Report-of-the-Committee-on-Infectious-Diseases-33rd-Edition-Paperback?srsltid=AfmBOoo28CqPlTyJj_iKiTejYoJfjWPSOKpW5Hs9A6sa-fXaPF3vXl8T.

[18] Fu P., Yan G., Li Y., Xie L., Ke Y., Qiu S., Wu S., Shi X., Qin J., Zhou J. (2024). Pertussis upsurge, age shift and vaccine escape post-COVID-19 caused by ptxP3 macrolide-resistant Bordetella pertussis MT28 clone in China. Clin. Microbiol. Infect..

[19] Lebel M.H., Mehra S. (2001). Efficacy and safety of clarithromycin versus erythromycin for the treatment of pertussis: A prospective, randomized, single blind trial. Pediatr. Infect. Dis. J..

[20] Langley J.M., Halperin S.A., Boucher F.D., Smith B., Pediatric Investigators Collaborative Network on Infections in Canada (PICNIC) (2004). Azithromycin is as effective as and better tolerated than erythromycin estolate for the treatment of pertussis. Pediatrics.

[21] Altunaiji S., Kukuruzovic R., Curtis N., Massie J. (2007). Antibiotics for whooping cough (pertussis). Cochrane Database Syst. Rev..

[22] Bortolussi R., Miller B., Ledwith M., Halperin S. (1995). Clinical course of pertussis in immunized children. Pediatr. Infect. Dis. J..

[23] Carlsson R.M., von Segebaden K., Bergstrom J., Kling A.M., Nilsson L. (2015). Surveillance of infant pertussis in Sweden 1998–2012; severity of disease in relation to the national vaccination programme. Euro Surveill..

[24] Bergquist S.O., Bernander S., Dahnsjö H., Sundelöf B. (1987). Erythromycin in the treatment of pertussis: A study of bacteriologic and clinical effects. Pediatr. Infect. Dis. J..

[25] Winter K., Zipprich J., Harriman K., Murray E.L., Gornbein J., Hammer S.J., Yeganeh N., Adachi K., Cherry J.D. (2015). Risk Factors Associated with Infant Deaths from Pertussis: A Case-Control Study. Clin. Infect. Dis..

[26] Van der Zee A., Schellekens J.F.P., Mooi F.R. (2015). Laboratory Diagnosis of Pertussis. Clin. Microbiol. Rev..

[27] Ebell M.H., Marchello C., Callahan M. (2017). Clinical Diagnosis of Bordetella Pertussis Infection: A Systematic Review. J. Am. Board Fam. Med..

[28] Gould I.M. (2009). Antibiotic resistance: The perfect storm. Int. J. Antimicrob. Agents.

[29] Muloiwa R., Kagina B.M., Engel M.E., Hussey G.D. (2020). The burden of laboratory-confirmed pertussis in low- and middle-income countries since the inception of the Expanded Programme on Immunisation (EPI) in 1974: A systematic review and meta-analysis. BMC Med..

[30] Heil J., Ter Waarbeek H.L., Hoebe C.J., Jacobs P.H., van Dam D.W., Trienekens T.A., Cals J.W., van Loo I.H., Dukers-Muijrers N.H. (2017). Pertussis surveillance and control: Exploring variations and delays in testing, laboratory diagnostics and public health service notifications, the Netherlands, 2010 to 2013. Euro Surveill..

[31] Xu Z., Wang Z., Luan Y., Li Y., Liu X., Peng X., Octavia S., Payne M., Lan R. (2019). Genomic epidemiology of erythromycin-resistant Bordetella pertussis in China. Emerg. Microbes Infect..

[32] Fu P., Zhou J., Yang C., Nijiati Y., Zhou L., Yan G., Lu G., Zhai X., Wang C. (2023). Molecular Evolution and Increasing Macrolide Resistance of Bordetella pertussis, Shanghai, China, 2016–2022. Emerg. Infect. Dis..

[33] Tozzi A.E., Gesualdo F., Rizzo C., Carloni E., Russo L., Campagna I., Villani A., Reale A., Concato C., Linardos G. (2020). A data driven clinical algorithm for differential diagnosis of pertussis and other respiratory infections in infants. PLoS ONE.

[34] Baxter R., Bartlett J., Fireman B., Lewis E., Klein N.P. (2017). Effectiveness of Vaccination During Pregnancy to Prevent Infant Pertussis. Pediatrics.

[35] Caro J.J., Getsios D., Payne K., Annemans L., Neumann P.J., Trindade E. (2005). Economic burden of pertussis and the impact of immunization. Pediatr. Infect. Dis. J..

[36] Heininger U. (2012). Pertussis: What the pediatric infectious disease specialist should know. Pediatr. Infect. Dis. J..

[37] Knapp J.K., Wilson M.L., Murray S., Boulton M.L. (2016). The impact of healthcare visit timing on reported pertussis cough duration: Selection bias and disease pattern from reported cases in Michigan, USA, 2000–2010. BMC Infect. Dis..

[38] Pandolfi E., Gesualdo F., Rizzo C., Russo L., Campagna I., Carloni E., Concato C., Linardos G., Villani A., Ciampini S. (2021). The impact of pertussis in infants: Insights from a hospital-based enhanced surveillance system, Lazio region, Italy, 2016 to 2019. Euro Surveill..

[39] Cox D.R. (1972). Regression Models and Life-Tables (with Discussion). J. R. Stat. Soc. Ser. B Methodol..

